# Between Psychopathology and Ideology: Challenges and Practices in Interpreting Young Extremists Experiencing Mental Illness in the Netherlands

**DOI:** 10.3389/fpsyt.2021.790161

**Published:** 2022-01-18

**Authors:** Floris Vermeulen, Maarten van Leyenhorst, Ineke Roex, Norah Schulten, Najib Tuzani

**Affiliations:** ^1^Political Science Department, Amsterdam Institute for Social Science Research (AISSR), University of Amsterdam, Amsterdam, Netherlands; ^2^Correctional Institutions Agency (Dienst Justitiële Inrichtingen), The Hague, Netherlands; ^3^Nuance Door Training & Advies, Deventer, Netherlands

**Keywords:** psychopathology, extremism, Netherlands, young people, professional

## Abstract

How violent extremism is interpreted among adolescents and young adults who experience a form of psychopathology can have far-reaching consequences for these youth and for society. A fundamental consideration here is the role that ideology and psychopathology play in radicalization and possibly related extremist violence. Risk management is challenged at various levels. This article seeks to contribute to academic and policy discussions on psychopathology and extremism by combining relevant insights from practices in the Netherlands. In this perspective article our aim is to stimulate awareness and research, on the basis of operational knowledge of the Dutch case, that helps professionals across the various domains of risk management with more expertise and the ability to better integrate and interact the concepts of psychopathology and ideology. We end with formulating hypotheses for further research.

## Introduction

How violent extremism is interpreted among adolescents and young adults who experience a form of psychopathology can have far-reaching consequences for these youth and for society. A fundamental consideration here is the role that ideology and psychopathology play in radicalization and possibly related extremist violence. Risk management is challenged at various levels. At the prevention level there are often no definitive guidelines on to what extent someone who displays extremist views and has a mental illness can be labeled a radical. And if an individual with a history of espousing extremist ideology commits an assault during a psychotic episode, is it still an extremist act?

An individual making extremist statements or showing extremist behaviors can have an underlying mental disorder, but its presence does not necessarily exclude the existence or relevance of other significant motives, personal or otherwise. The individual's environmental context can be an equally important factor for explaining behavior. Professional assessment takes place across various domains, each with its own specific albeit overlapping dilemmas. Legally, the question is to what extent certain behaviors and/or ideas can be attributed to the individual. Criminal justice partners, such as prisons and probation officers, tend to focus on how to reduce the chance of recidivism.

There is great academic interest in the underlying factors toward an individual's process of violent extremism and what possible role psychopathology plays in that process. Currently, however, we lack a clear framework for adequate assessment in practical cases. To arrive at a well-founded assessment and approach, both concepts, psychopathology and ideology, must be better integrated and accurately interpreted across the various domains. Ultimately achieving this requires more research, since many questions cannot yet be answered by academic research alone. Once they have better understanding of both concepts, highly trained specialists can then arrive at an integrated risk assessment.

This perspective article seeks to contribute to the academic and policy discussions on psychopathology and extremism using insights from practices in the Netherlands, especially from the domain of criminal law. We are aware of the lack of academic consensus on the terms radicalization and extremism (1), in this article we use these terms to discuss cases in which ideological violent elements are perceived or established. We begin by briefly examining the relationship between psychopathology and violent extremism from an academic perspective. Then, we focus on Dutch practices as experienced by ourselves as professionals in specific (criminal) cases and, within them, the recurring challenges. The article concludes by proposing a research agenda based on our practical knowledge. Our aim is not to test our assumptions and experiences, but provide a research perspective that ultimately lead to equipping professionals across the various domains of risk management with more expertise and the ability to better integrate and interact the concepts of psychopathology and ideology. We end with the formulation of hypotheses for further research.

## Academic Research on the Relationship Between Psychopathology and Violent Extremism

Research on violent extremism shows that risks factors associated with extremist violence differ from those associated with regular violent behavior (2, 3). Instruments have accordingly been developed to assess risk of violent extremism, although a psychopathological assessment is not automatically intrinsic to them. These assessment instruments can also be used by professionals who have experience in the field and with risk analysis, even though they themselves are not mental healthcare professionals.

Recent research shows that the presence of a mental illness is in itself not a direct predictor for extremism. It is therefore important that extremism, as a societal problem, will not be unnecessarily medicalized (4, 5). It is, however, the case that individuals with a mental illness can also espouse an extremist ideology, and that the presence of one does not automatically exclude the other (6). It depends on the individual case if and how mental illness plays a role in someone's process toward violent extremism (7). When present, the combination of psychopathology and extremism is very complex to analyze and assess and it requires multiple professionals and a holistic assessment. Recent scientific studies also indicate that individuals who are referred or monitored because of concerns about potential violent extremism should be examined to assess if mental health problems are present. All forms of psychopathology can occur in extremists across all ideological backgrounds. It remains difficult, however, to draw one overarching conclusion from publicly available research. The percentage of registered cases of mental health issues among different groups of extremists in different countries ranges from 0% up to 57%. The great variation in this range is due to inconsistencies across studies. Researchers have examined different subgroups in different contexts, had access to different data sources and methods, and interpreted forms of psychopathology differently (5).

Certain forms of psychopathology can be elevated in some types of extremists, although drawing any such conclusions is hard because we often lack a basis of comparison, statistical or otherwise, with control groups. For example, several studies have identified mental health issues in a significant number of lone actor extremists (8–10). In one sample of lone actors, an analysis of descriptive statistics showed that prevalence rates of schizophrenia, delusional disorder, and autism spectrum disorders seemed elevated compared to the general population base rate (11). Research does also indicate that prevalence rates of mental illness in samples of extremists who tend to operate in groups are less high than in lone actor samples, possibly because of the different roles violent extremists have in groups (12, 13). Nonetheless, researchers have identified specific mental health problems among foreign fighters and jihadists (e.g., psychotic disorders and PTSD) (14, 15); right-wing extremists (e.g., substance use) (16); and suicide bombers (e.g., depression and suicidality) (17). How these mental disorders constellate with many other (group) mechanisms, such as social isolation, peer pressure, and seeking for significance, is characterized by heterogeneity and thus warrants tailored assessments and case-management (5, 18).

Adolescents who hold extremist views and experience psychological issues are part of a psychologically vulnerable target group in a turbulent life phase (19, 20). Adolescence is accompanied by a search for identity (21, 22), and this search is identified as one of the important drivers among extremist groups (23, 24). Prevalence rates of specific mental disorders, such as depression and ADHD, can also differ between childhood, adolescence and adulthood phases (19). Whether psychopathology plays a different role in the radicalization process of adolescents compared to adults has not yet been studied extensively. Oppetit et al. (20) do show that adolescents may display different psychological pathways toward violent extremism than adults. For example, in their sample, radicalized adolescents more often had a history of self-harm. However, prevalence rates of depressive symptoms and substance use and addiction were not significantly different between adolescents and adults.

Recent research also shows that different forms of extremism can be assessed differently when it comes to the role that ideology and/or psychopathology play in them (25–29). Islamist-inspired extremism is likelier to be referred to as terrorism by security agencies, the media, and public opinion than violence inspired by right-wing or left-wing extremism (30). Muslims are generally described more negatively by the media than other groups and likelier to be associated by the media with violent extremism than other groups with the risk that right-wing extremism is more likely to be interpreted as psychopathological (25–29). Studies also report that while Islamist-inspired extremist acts are more likely to be labeled as terrorism in the media, those who commit far-right-inspired extremist acts are not more likely to be labeled mentally ill than individuals from other ideological groups (30).

## Psychopathology and Extremism in Dutch Practice

It is the responsibility of various bodies in the Netherlands to assess and follow up on radicalization and extremism cases. In the Netherlands periodic consultations occur between local care and security partners to discuss such cases. They may involve individuals who are not yet criminally charged, but about whom authorities have radicalization concerns. In general, once an individual is suspected or convicted of a criminal act, their case enters the criminal justice system. The public prosecutor can then prosecute the suspect for a terrorist offense and take them into preventive custody. For terrorism-related cases, this usually means placement within a terrorism ward, though individuals prosecuted for terrorism are also placed in a regular ward. There are multiple reasons for this, such as age, arguments to prevent further radicalization, and concerns of psychopathology.

The potential or actual presence of psychopathology in such cases not only has consequences for the detention facility. Experiencing a form of psychopathology may lead the individual to receive a shorter or different sentence. It can also impact their treatment and guidance, the domain in which social reintegration takes place (such as healthcare or criminal law), and thus on the professionals working on the case. If proven terrorist intent is an aggravating factor in a sentence, the presence of psychopathology can also shorten or soften punishment. Psychopathology can therefore serve as a mitigating circumstance. In some cases, a judicial decision focused on care and treatment may be a shorter duration than a prison sentence. Declaring someone as being beyond treatment (a decision that may be hastened in cases of feigned psychopathology) can mark the end of a criminal law framework that would allow a convicted individual to return to society relatively quickly.

The assessment of the extent to which an extremism case (also) involves (integration of) psychopathology is made by different professionals at different times in the Netherlands. Preparing for the substantive hearing of a criminal case, multiple agencies conduct research into issues, such as risk of recidivism or ideological perception. Partly drawing from these investigations, the judiciary creates an image on which its judgment is based. The judicial decision has consequences not only on the circumstances under which someone is detained, but for the entire process, from the moment of the decision up to and including the process of reintegration into society. How a convicted person is treated, their prison trajectory, transfer to aftercare partners, and much else depend on the weighing and integration of criminogenic factors.

Although assessments from multiple parties can together provide a good picture of an individual, nuances and the weight attributed to them can have a major effect on a case's settlement and, consequently, on safety for society. Moreover, differing perspectives and expertise are brought to bear on the same case and, on that basis, advice is given on further follow-up and risk management. Although mutual consultation takes place, it does not always lead to consensus on decisive risk factors nor, therefore, on the most appropriate approach.

The risk factors associated with violent extremism, as mentioned in the theoretical framework, also require specific assessment instruments and specialized knowledge and experience. This is an important reason why the criminal law chain in the Netherlands includes trained professionals. Outside the chain, some municipalities also have specially trained and assigned aftercare staff. Risk assessment instruments and interpretation methods are used in the formulation of advice among the various partners. This does not mean, however, that in Dutch practice, the concepts of psychopathology and ideology are always examined extensively or in conjunction with each other.

Two of the most used instruments in Europe regarding the assessment of violent extremism, the Violent Extremist Risk Assessment 2 Revised (VERA-2R) and the Extremism Risk Guidelines (ERG22+) require a user to be able to identify certain ideological positions. Concepts such as ideological grievances, anger toward the government, and us-vs.-them thinking are indeed part of these risk assessment instruments, but underlying ideological commitment are not meaningfully examined by these instruments. Similar precautions apply in the integration of psychopathology. Although these instruments support the professional in making assessments about the possible presence or absence of psychopathology, the assessments are not necessarily conducted by mental health professionals. Moreover, these specific instruments examine the presence and relevance of psychopathology only superficially. The integration of both concepts, psychopathology and an extremist ideology, is also not included in these instruments, predominantly due to a lack of evidence based knowledge regarding the way the concepts (possibly) interact with each other.

The majority of professionals in the Dutch healthcare chain has no specific expertise on terrorism nor access to risk assessment tools for violent extremism (3). Experience shows that many healthcare professionals are also unaware of the existence of the specialized departments of several agencies with which they can collaborate. An (exclusive) emphasis on psychopathology can therefore, unjustifiably, lead to less attention on an individual's ideology. It can lead to a situation where someone suspected or convicted of a crime no longer receives mentoring from services specialized in extremism, but merely gets mentoring and aftercare from partners with a primary focus on and expertise in psychopathology.

Ideology is a complex, personal, easily concealed, and therefore more difficult phenomenon to establish than objectified investigative information and validated forensic diagnostics. This was manifested in the case of Malek F., who attacked several people in The Hague on Liberation Day 2018. On an appeal, the public prosecutor's office demanded F. to be acquitted of acting with a terrorist intent, even though the office included this intent on the original indictment. In this case, there was contradicting advice from the involved authorities, with one emphasizing psychopathology and another indicating the additional evidence of adhering to an extremist ideology. The public prosecutor's office chose not to press charges for terrorism because, in their words, adherence to an extremist devotion and any impact of this ideology on the criminal behavior were very difficult to prove.^1^ The final outcome was a conviction of various attempts of manslaughter and not for a (more aggravated) terrorist offense.

## Experiences From Dutch Practice and Differences Between Individuals and Groups

It goes beyond the scope of this article to provide a systematic description of how Dutch practices actually play out. This article therefore introduces experiences from several stakeholders in the Dutch counter-terrorism field in order to learn from operational experience to stimulate debates on future research. The two co-authors who work for NTA,^2^ Najib Tuzani and Ineke Roex, have been ideological experts in several criminal cases. Maarten van Leyenhorst is senior program official on the approach of radicalization and extremism for the Dutch Correctional Institutions Agency. This program offers coordination and monitoring on all risks and reintegration trajectories of detainees related to extremism. In this section, we share our observations focused specifically on differences according to gender, types of extremism, and age.

Among women, we have sometimes witnessed a bias among professionals who evaluate the extent to which ideology is a motivating factor in violent extremism. Some women may also exaggerate expressions of naivety, victimization, and possible trauma (as a result of their experience in a conflict zone). This can lead to a situation in which a woman's personal choices that have arisen from extremist ideology are insufficiently incorporated and thus accounted for in risk management. Insights into ideological risks may thus fade into the background. This potentially relevant information may go untapped even when the women's experiences include significant circumstances, such as many years of residence in Syria or residence there upon the fall of the last ISIS stronghold; cohabitation with female fighters; involvement in other extremist networks; a history of actively spreading propaganda; and premeditated deception after return to the Netherlands.

In cases of right-wing and left-wing extremism, much attention goes to frontend counseling, aftercare support, and proper embedding in care facilities (for example, to help with autism, cognitive skills, companionship-building, and influence). Such cases are still too often judged as “being crazy” or “losers,” whereby attention for ideological legitimation of violence threatens to fade into the background. This can lead to psychopathology becoming overly dominant in risk management decisions. A lot of discussion takes place between the agencies involved, but contributions clearly emerge from their respective specializations.

For example, there are known cases that someone is or has been involved in a right-wing extremist group based on personal impressionability. This suggestibility then comes first in risk management, while the interpretation and approach of a person who has also become involved in Islamic-inspired extremism through personal suggestibility is more often explained and guided from an ideological perspective. An important question here is what the explanation is for the difference in interpretation. Does this have to do with signaling and analysis on the advice side (e.g., due to lack of insight into/knowledge of what is now deviant right-left extremist language, after all this is often normalized) or due to (expected) limitations of or limited insight into the supply side of such guidance? Is there sufficient (view of) supply to provide right-wing extremism (also with regard to the left, anti-government, eco, etc.) with appropriate ideological guidance, especially now that such extremist casuistry is increasing? In practice, there seems to be a large gray area around the right and left with regard to what is allowed vs. behavior and statements that should be judged as extremist. Such a gray area offers more room to investigate alternative criminogenic/risk-increasing (including psychopathological) factors. With regard to religious extremism, there is a chance that the (ideological) contradiction between norm and deviation (violence) will be assessed in a more absolute (and therefore recognizable) way, with psychopathology being regarded as a side issue rather than a factor that must be integrated into the overall risk picture.

With adolescents and young adults, the Dutch approach is often to focus on behavioral change, positive behavior and educational development. Getting insight into ideological risks requires however specific knowledge. In adolescents, statements that may be extremist, such as those glorifying violence, are often dismissed as signs of social development, search for identity, psychopathology, or just acting tough. Although those features may also play a role in making such statements, they can also actually contain an ideological legitimation of violence. By focusing primarily, if not exclusively, on adolescent development, professionals can lose sight of the possibility of extremist violence legitimized by an extremist ideology. In Dutch criminal law, several examples involve individuals whom the police and judiciary system have monitored since adolescence because of potential terrorism concerns. A well-known case involves Wail el-A., who was convicted in 2016 for attempting to travel to Syria and Iraq and placed in a juvenile detention center. In 2018, he was arrested as part of the cell known as the Arnhem group, which was preparing to commit an attack in the Netherlands.

In the Netherlands, we thus see differences in assessment and interpretation on an individual level, which can differ by gender and age, and on a group level, which can differ according to types of extremism. This can hold true even when relatively similar risk profiles seem to be present. These inconsistencies seem to be the result of existing ideas in society about causes and manifestations of violent extremism as well as a lack of knowledge regarding specific options for supervision and treatment for left-wing and right-wing extremism.

## Discussion and Research Agenda

There still seems to be too little attention for the integration of, and interaction between, psychopathology and ideology when it comes to the assessment and follow-up of terrorism cases. By and large, risk management currently dichotomizes the concepts. If psychopathology is found to be a decisive factor for an extremist ideology and leads to risk management on the part of care facilities then signaling, treatment, and risk management become primarily the domain of those care professionals. This integration of psychopathology and ideology is crucial for an optimal risk assessment in such cases.

What is needed is thorough interpretation and analysis, conducted as methodically as possible This should be aimed at understanding the impact of both psychopathology and ideology on an individual's actions. In this way, justice can be administered in a well-founded manner, resulting in a more appropriate follow-up of the criminal justice system, treatment, mentoring, and aftercare. To achieve this, we need more research into ways to integrate psychopathology and ideology as drivers of violent extremism. Research should span multiple spheres. That includes the conceptual; the explanatory–asking, for example, how both concepts can help explain extremism in the population of violent extremists; and the uptake (more and better combined advice from different fields of expertise). Such research should lead to further development of specific risk assessment instruments of ideology in relation to psychopathology. It would require extensive primary-source research into the individual who has committed an extremist act, the act itself, and environmental context.

Further research is also needed to understand why psychopathology and ideology are interpreted and assessed differently in individual cases of violent extremism as well as in different forms of extremism. On the basis of our Dutch experience we foresee, in terms of gender, that ideology plays less of a role in the assessment of violent women compared to men. In terms of ideology, we expect that right-wing and left-wing extremists, in comparison with Islamic-inspired extremists, are more often assessed from a psychopathological perspective ignoring ideological elements. And in terms of age we anticipate extremist behavior among adolescents and young adults to be evaluated less often as something ideological compared to adult extremists. Further research in different contexts is needed to further study and test these generated hypotheses.

Although assessment instruments provide tools for professionals to evaluate the possible presence or absence of psychopathology, as mentioned above, mental health professionals are not necessarily involved and these instruments only superficially examine possible psychopathology. More importantly, the actual integration of psychopathology and ideology as drivers of criminal behavior has not yet been assessed in a sufficiently methodical way. Doing so would require more extensive consultation between the agencies involved or a professional framework equally specialized in both concepts.

## Data Availability Statement

The raw data supporting the conclusions of this article will be made available by the authors, without undue reservation.

## Author Contributions

All authors listed have made a substantial, direct, and intellectual contribution to the work and approved it for publication.

## Conflict of Interest

IR and NT were employed by company Nuance Door Training & Advies. The remaining authors declare that the research was conducted in the absence of any commercial or financial relationships that could be construed as a potential conflict of interest.

## Publisher's Note

All claims expressed in this article are solely those of the authors and do not necessarily represent those of their affiliated organizations, or those of the publisher, the editors and the reviewers. Any product that may be evaluated in this article, or claim that may be made by its manufacturer, is not guaranteed or endorsed by the publisher.

## Footnotes

^1^ https://uitspraken.rechtspraak.nl/inziendocument?id=ECLI:NL:RBDHA:2019:7308

^2^NTA is an independent consultancy and research agency that contributes to the (person-oriented) approach to radicalization and violent extremism.
